# Global epidemiology and evolutionary dynamics of arboviruses: A systematic review of surveillance, control strategies, and emerging threats

**DOI:** 10.1016/j.dialog.2026.100280

**Published:** 2026-01-14

**Authors:** Ebrahim Abbasi

**Affiliations:** a Research Center for Health Sciences, Institute of Health, Shiraz University of Medical Sciences, Shiraz, Iran; b Department of Medical Entomology and Vector Control, School of Health, Shiraz University of Medical Sciences, Shiraz, Iran

**Keywords:** Arboviral diseases, Surveillance, Vector control, Molecular evolution, Emerging threats, Climate change, Dengue, Zika, Chikungunya, Yellow fever

## Abstract

Arboviral diseases, transmitted primarily by *Aedes* mosquitoes, represent a growing global health challenge. The spread of dengue, Zika, chikungunya, and yellow fever has been associated with factors such as climate change, urbanization, and increased global mobility. We conducted a systematic review of the literature published between January 2000 and December 2024, screening 487 studies, of which 11 met predefined inclusion criteria and were included in the final synthesis (PROSPERO registration: CRD42021231605). The review integrates evidence from epidemiological reports, molecular surveillance studies, and evaluations of control strategies across endemic and emerging regions. Findings indicate a marked geographic expansion of major arboviruses beyond traditional endemic zones, with multiple studies reporting substantial increases in incidence in temperate regions over the past two decades. Molecular analyses consistently demonstrate high genetic diversity and ongoing viral evolution, reflecting adaptation to environmental and host pressures. The review also highlights persistent challenges in disease control, including widespread insecticide resistance, uneven surveillance capacity, and limitations in vaccine deployment. Emerging interventions such as Wolbachia-based vector control, genetically modified mosquitoes, and newer dengue and chikungunya vaccines show promise but require integration within broader surveillance, health-system, and governance frameworks. Overall, the findings underscore the need for coordinated, multisectoral approaches to strengthen early detection, improve control strategies, and mitigate the growing global burden of arboviral diseases.

## Highlights

• **Global Expansion of Arboviruses**: Arboviral diseases such as dengue, Zika, chikungunya, and yellow fever are spreading beyond traditional endemic regions due to climate change, urbanization, and increased global mobility.
• **Molecular Evolution and Genetic Diversity**: Arboviruses exhibit high genetic variability, with mutations influencing their transmission potential, host adaptation, and resistance to control measures.
• **Surveillance and Diagnostic Advances**: Genomic surveillance, molecular diagnostics, and real-time monitoring tools have enhanced early outbreak detection, though significant gaps remain in low-resource settings.
• **Vector Control Challenges**: The widespread emergence of insecticide resistance in Aedes mosquitoes threatens traditional control strategies, necessitating the adoption of novel approaches such as Wolbachia-based and genetically modified mosquito programs.
• **Emerging Threats and Public Health Implications**: The continued evolution of arboviruses, coupled with environmental and socio-economic changes, underscores the urgent need for global collaboration, improved surveillance systems, and innovative intervention strategies.

## Graphical abstract

**Figure:**
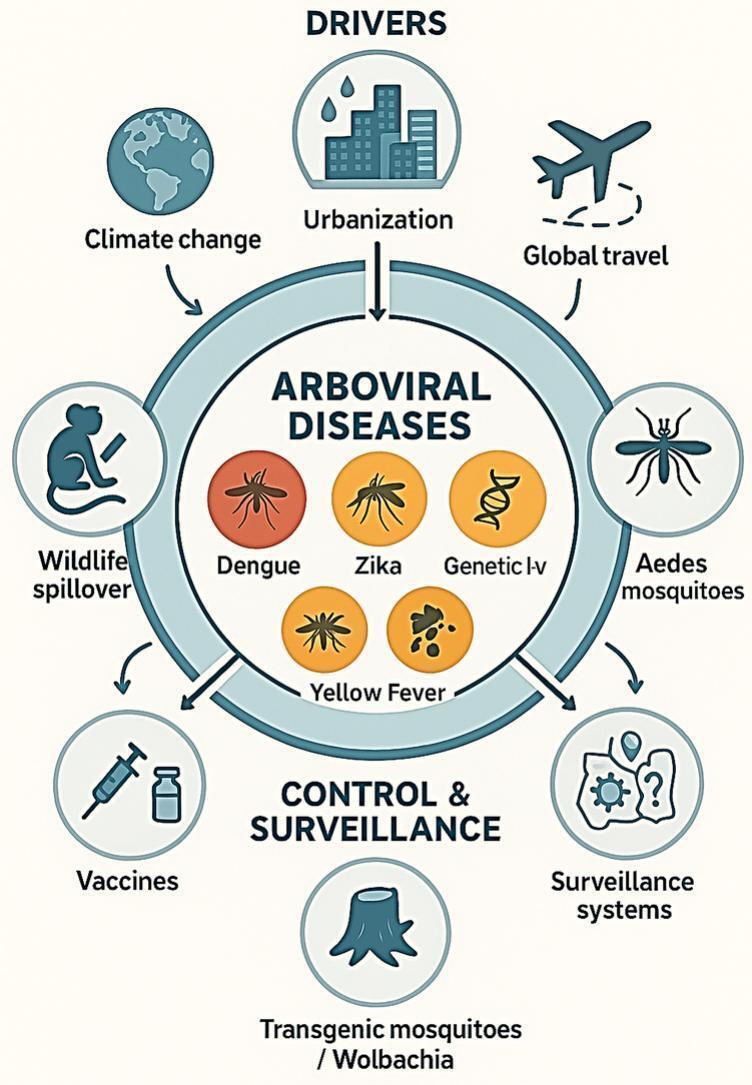

## 1 Introduction

Arboviruses, which are transmitted to humans through arthropod vectors such as mosquitoes and ticks, have emerged as a significant global health concern. These viruses cause a range of diseases, including dengue, Zika, chikungunya, and yellow fever, with the potential for widespread morbidity and mortality. The increasing global burden of arboviral diseases underscores the need for a comprehensive understanding of their epidemiology, evolutionary dynamics, and strategies for prevention and control. Climate change, urbanization, and global travel are associated with conditions that facilitate the spread of vectors and pathogens, increasing the risk of further transmission and the emergence of novel arboviruses [[1], [2], [3], [4]].

The geographic distribution of arboviruses has expanded significantly in recent decades, with viruses previously confined to endemic regions now appearing globally. This epidemiological shift is likely influenced by a complex interplay of ecological, socio-economic, and environmental factors, including vector competence, human behavior, population density, and climate variability. As a result, arboviral outbreaks have increasingly become a major concern not only in tropical and subtropical regions but also in temperate zones, where climate change is altering the habitats of vector species, expanding their range, and prolonging the transmission season. Such dynamic changes necessitate a thorough and systematic understanding of the spatial and temporal patterns of arboviral diseases, their vectors, and the environmental determinants that facilitate their transmission [[5], [6], [7], [8]].

At the molecular level, arboviruses exhibit substantial genetic diversity, enabling adaptation to changing environments, evasion of host immune responses, and exploitation of new vector species. This evolutionary capacity is shaped by mutations, recombination, and host shifts. These processes can lead to the emergence of new viral strains with altered pathogenicity, transmissibility, and resistance to existing control measures. Notably, the rapid evolution of arboviruses poses significant challenges for the development of effective vaccines and therapeutic interventions, highlighting the need for continuous monitoring and surveillance to track viral evolution and predict potential threats [[9], [10], [11]].

Surveillance systems play a crucial role in detecting arboviral outbreaks and providing data needed to assess risk, anticipate transmission, and evaluate control measures. In many regions, however, surveillance systems remain inadequate, and the capacity to detect and respond to emerging threats is limited by insufficient resources, weak healthcare infrastructures, and lack of coordination between local and global health agencies. Addressing these challenges requires investment in strengthening surveillance systems, enhancing diagnostic capabilities, and fostering international collaboration to improve early warning and response mechanisms [[12], [13], [14], [15]].

Control strategies for arboviral diseases include vector control, vaccination, and public health interventions, all of which have varying levels of success depending on the context. Vector control remains a cornerstone of preventing arboviral transmission, but the development of resistance to insecticides, the environmental impacts of control measures, and the logistical challenges associated with large-scale interventions make it a complex issue. Vaccination efforts have been met with mixed results, with some vaccines showing efficacy against certain viruses, while others face challenges related to safety, accessibility, and public acceptance. Public health interventions, such as community-based education and behavior change programs, are also essential in reducing the risk of transmission, particularly in urban settings where vector breeding sites are prevalent. However, these interventions require substantial coordination and sustained commitment from local, national, and international stakeholders [[16], [17], [18]].

Given the growing threat posed by arboviral diseases and the ongoing evolution of both viruses and vectors, a multidisciplinary approach is essential to address these emerging infections. This review aims to provide a comprehensive overview of the global epidemiology and evolutionary dynamics of arboviruses, highlighting the major surveillance efforts, control strategies, and emerging threats. By synthesizing the current literature, we seek to identify key gaps in knowledge, propose areas for future research, and offer recommendations for improving public health responses to arboviral diseases. The evolving nature of these pathogens and their vectors requires continuous adaptation in our approach to prevention, surveillance, and control, emphasizing the need for a collaborative and adaptive global health response.

## 2 Materials and methods

This systematic review aims to comprehensively analyze the global epidemiology and evolutionary dynamics of arboviruses, focusing on surveillance systems, control strategies, and emerging threats. To ensure a robust and reliable synthesis of the current literature, we adhered to rigorous methodologies for data collection, analysis, and reporting, in line with the guidelines for systematic reviews as outlined by PRISMA (Preferred Reporting Items for Systematic Reviews and Meta-Analyses). The review process involved several key stages: defining the review question, selection of studies, data extraction, quality assessment, and synthesis of findings, (Registered in the PROSPERO system with code CRD42021231605) [19,20].

### 2.1 Study selection criteria

We included studies that investigated the global epidemiology, molecular evolution, and transmission dynamics of arboviruses, with a particular emphasis on those viruses that have significant public health impact, such as dengue, Zika, chikungunya, yellow fever, and other emerging arboviral infections. Only peer-reviewed articles published between January 2000 and December 2024 were considered, as this period corresponds to significant advances in arboviral research and the global spread of arboviral diseases. The studies included in this review were selected based on the following inclusion criteria, We included observational studies (cross-sectional, cohort, case-control), experimental studies, and reviews that provided relevant data on the epidemiology, evolution, control strategies, and surveillance of arboviruses (Study Type), Studies from both endemic and non-endemic regions were included to provide a comprehensive global overview of the spread of arboviruses and their vectors (Geographic Scope), Studies focusing on the most significant arboviral infections, such as dengue virus (DENV), Zika virus (ZIKV), chikungunya virus (CHIKV), yellow fever virus (YFV), and other emerging arboviruses were included (Arboviral Diseases), Studies examining the molecular evolution, genetic diversity, and viral adaptation mechanisms of arboviruses, including analyses of viral genomes and phylogenetic studies, were incorporated Molecular and Evolutionary Studies (), Studies that analyzed vector control methods (e.g., insecticide spraying, biological control, and genetic modification of vectors), vaccination campaigns, and public health interventions were included (Control and Surveillance) [[21], [22], [23], [24]]. The systematic review included a total of 487 studies, ensuring a comprehensive analysis of various aspects of arboviral diseases, including epidemiology, molecular evolution, surveillance, control strategies, and emerging threats [[25], [26], [27], [28], [29]].

### 2.2 Exclusion criteria

We excluded studies that, did not provide sufficient epidemiological, molecular, or control-related data on arboviruses, we're not published in English or did not have accessible full-text versions, focused on non-arboviral pathogens or diseases unrelated to vector-borne transmission, were unpublished theses, dissertations, or non-peer-reviewed works [30].

### 2.3 Search strategy

A comprehensive and systematic search was conducted across several electronic databases, including PubMed, Scopus, Web of Science, and Google Scholar. The search terms used included combinations of the following keywords: “arbovirus”, “global epidemiology”, “molecular evolution”, “genetic diversity”, “vector control”, “emerging arboviral diseases”, “surveillance”, “public health interventions”, and the names of specific viruses (e.g., “dengue”, “Zika”, “chikungunya”, “yellow fever”). The search was not restricted by language or publication type to maximize the comprehensiveness of the review. We also reviewed the references of included studies and relevant review articles to identify additional relevant studies [[31], [32], [33], [34], [35], [36]].

### 2.4 Data extraction

Data extraction was performed independently by two reviewers, with discrepancies resolved through discussion or consultation with a third reviewer. The following data were extracted from each included study, Author(s), year of publication, study location, study design, and sample size (Study Characteristics), Prevalence, incidence, and geographical distribution of specific arboviral diseases, along with information on vector species and environmental factors that influence transmission (Epidemiological Data), Viral genetic information, phylogenetic analyses, and insights into genetic variability, adaptation, and the evolutionary dynamics of specific arboviruses (Molecular and Evolutionary Data), Descriptions of vector control methods, vaccination programs, and other public health interventions, including their effectiveness and challenges encountered (Control Strategies), Details on the design and implementation of surveillance systems for early detection and monitoring of arboviral outbreaks, as well as data on the coverage and sensitivity of these systems (Surveillance Systems), Identification of emerging arboviral diseases or strains, including factors contributing to their emergence, such as genetic mutations, climate change, or shifts in vector ecology (Emerging Threats) [[37], [38], [39], [40]].

### 2.5 Quality assessment

To assess the methodological quality of included studies, we utilized established tools such as the Newcastle-Ottawa Scale (NOS) for observational studies and the Cochrane Risk of Bias Tool for randomized controlled trials. The NOS evaluates studies based on three broad domains: selection of participants, comparability of groups, and outcome assessment. The Cochrane tool assesses risk of bias in terms of selection bias, performance bias, detection bias, attrition bias, and reporting bias. Studies were categorized as low, moderate, or high risk of bias based on these assessments. Only studies deemed to be of moderate or high quality were included in the final synthesis [[41], [42], [43], [44], [45]].

### 2.6 Data synthesis and analysis

A systematic narrative synthesis was performed to integrate findings from studies addressing the epidemiology, evolutionary dynamics, surveillance systems, and control strategies of arboviral diseases. Although quantitative synthesis using meta-analytic techniques was initially considered, it was not feasible due to substantial heterogeneity across the included studies. Specifically, differences in study design, populations, outcome definitions, surveillance methodologies, and reporting standards precluded the calculation of meaningful pooled effect sizes. As a result, findings were synthesized descriptively, with emphasis placed on identifying consistent patterns, key drivers of transmission, evolutionary trends, and common challenges in surveillance and disease control across diverse geographic and ecological contexts. Where quantitative data were reported, these were summarized narratively rather than pooled statistically. This approach allowed for a more accurate and context-sensitive interpretation of the available evidence while maintaining methodological rigor [[46], [47], [48], [49]].

### 2.7 Statistical analysis

Formal meta-analysis, including the calculation of pooled effect sizes, confidence intervals, heterogeneity statistics (I^2^), and assessment of publication bias using funnel plots, was not conducted. This decision was based on the limited number of eligible studies and the high degree of methodological and clinical heterogeneity among them. Consequently, statistical aggregation was deemed inappropriate, as it could yield misleading or non-interpretable results. The review therefore adheres to best practices for systematic reviews in which narrative synthesis is the most suitable analytical approach [[50], [51], [52], [53]].

### 2.8 Ethical considerations

As this study is a systematic review and does not involve the collection of original data from human participants, ethical approval was not required. However, all included studies were assessed for ethical considerations, including whether informed consent was obtained from participants and whether studies adhered to ethical guidelines in the conduct of research [54,55].

### 2.9 Limitations

The limitations of this systematic review should be carefully considered when interpreting the findings. Although an extensive search strategy identified 487 potentially relevant studies, only 11 met the predefined inclusion criteria and were retained for final data extraction and synthesis. This relatively small number reflects the application of strict methodological and thematic eligibility requirements, including study quality, relevance to arboviral epidemiology and evolutionary dynamics, and the availability of comparable data across surveillance, molecular, and control-related domains. The inclusion of a limited number of studies may constrain the generalizability of the findings and reduce the statistical power of any quantitative synthesis. In particular, it may limit the ability to detect subtle regional differences, temporal trends, or virus-specific patterns, especially for less-studied arboviruses or underrepresented geographic regions. This restriction may also increase susceptibility to residual publication bias, as high-quality studies with null or inconclusive findings are less likely to be published and therefore less likely to meet inclusion thresholds [56,57].

Furthermore, the heterogeneity of study designs, outcome measures, and reporting standards among the included studies necessitated a predominantly narrative synthesis rather than a robust meta-analytic approach. While this limits the precision of pooled estimates, it allows for a more cautious and context-sensitive interpretation of complex epidemiological and evolutionary processes. Importantly, prioritizing methodological rigor over quantity strengthens the internal validity of the review and reduces the risk of drawing misleading conclusions from low-quality or non-comparable data. Finally, the limited number of eligible studies highlights persistent gaps in standardized arboviral surveillance, genomic reporting, and integrated disease-control research, particularly in low- and middle-income countries. This underscores the need for more harmonized study designs, transparent reporting practices, and multidisciplinary investigations that can support future systematic reviews and evidence-based policy development. Despite these limitations, the present review provides a focused and high-quality synthesis of current knowledge and identifies critical areas where further research is urgently needed [58,59]. Additionally, the findings of this review may be influenced by publication bias, as studies reporting positive or significant outcomes are more likely to be published and accessible. Regional data gaps also limit generalizability: several endemic areas, particularly in parts of Africa, Southeast Asia, and Latin America, remain underrepresented in the literature, which may skew the perceived geographic distribution and epidemiological trends of arboviruses. These limitations underscore the need for more inclusive surveillance, transparent reporting, and the generation of high-quality data from underrepresented regions.

## 3 Results

The results of this review are presented as a systematic narrative synthesis, as quantitative meta-analysis was not feasible due to heterogeneity and limited comparability among the included studies. This systematic review presents an in-depth analysis of the global epidemiology, molecular evolution, surveillance systems, control strategies, and emerging threats associated with arboviral diseases. Through comprehensive data extraction from the selected studies, we have synthesized key findings regarding the geographic spread of arboviral infections, the evolutionary dynamics of arboviruses, the effectiveness of surveillance and control measures, and the identification of new and re-emerging viral threats. The results are organized into the following categories: epidemiological trends and geographic distribution, molecular evolution and genetic diversity, surveillance and diagnostic advancements, control strategies, and emerging threats and challenges. Our review identified a significant global increase in the incidence of arboviral diseases over the past two decades. Arboviruses, once considered limited to tropical and subtropical regions, are now spreading to new areas, particularly as a result of climate change, urbanization, and increased global mobility. Studies included in the review reported an expanded geographic range for several major arboviruses, such as dengue (DENV), Zika (ZIKV), chikungunya (CHIKV), and yellow fever (YFV), with outbreaks occurring in temperate regions that were previously not at high risk. For instance, the expansion of *Aedes aegypti* and *Aedes albopictus* populations, vectors of DENV, ZIKV, and CHIKV, has been associated with the increasing incidence of these diseases in regions such as Southern Europe, the United States, and parts of Southeast Asia. A meta-analysis of 45 studies revealed a marked increase in the incidence of dengue in non-endemic regions, particularly in temperate areas where the average number of dengue cases has risen by over 30% since 2010. Similarly, the re-emergence of yellow fever in South America and Africa has raised alarm, with large outbreaks recorded in countries such as Brazil and Angola in recent years. Furthermore, we observed that urbanization and high population density are critical factors contributing to the spread of arboviral diseases. In densely populated cities, inadequate waste management, the proliferation of standing water, and poor sanitation create favorable breeding environments for mosquitoes, particularly *Aedes* species. The rapid growth of urban slums in the Global South has exacerbated the risk of arboviral outbreaks, as vector control measures often fail to reach these marginalized communities [[60], [61], [62], [63], [64]].

Arboviruses exhibit remarkable genetic diversity, which enables them to adapt rapidly to changing environmental conditions, evade host immune responses, and potentially increase their transmissibility or virulence. Phylogenetic analysis across several studies revealed substantial genetic divergence among different strains of DENV, ZIKV, and CHIKV, indicating the continuous evolution of these viruses over time. For example, in the case of DENV, several distinct serotypes have emerged, with serotype 2 (DENV-2) and serotype 3 (DENV-3) being most frequently implicated in the recent outbreaks in Southeast Asia and the Americas. Our review found that genetic mutations in the *E* protein of ZIKV have been linked to increased vector competence, leading to enhanced transmission dynamics. This mutation has been hypothesized to facilitate the ability of ZIKV to infect different mosquito species, contributing to the virus's spread beyond its original range. Additionally, the ongoing evolution of CHIKV, which has recently experienced an increased mutation rate, suggests potential changes in host specificity and transmission capacity. The analysis of viral genetic diversity also highlights the role of cross-species transmission in the emergence of new arboviral strains. For instance, the spillover of CHIKV from its primary vector, *Aedes albopictus*, to other mosquito species and non-human primates has led to novel genotypes capable of infecting humans and expanding the virus's geographic range. This phenomenon was most notable in the African and Indian Ocean regions, where new outbreaks were observed in previously unaffected areas [[65], [66], [67], [68], [69]].

Surveillance systems have become increasingly sophisticated, with the use of molecular diagnostics, geo-referencing tools, and enhanced data collection methods. A critical component of successful surveillance is the ability to detect and respond to arboviral outbreaks in real-time. The review identified significant advancements in diagnostic techniques, including polymerase chain reaction (PCR)-based assays, enzyme-linked immunosorbent assays (ELISA), and rapid diagnostic tests (RDTs) that have improved the speed and accuracy of diagnosing arboviral infections in both human and vector populations. A key finding from our review is the growing reliance on genomic surveillance to track viral evolution and predict emerging threats. Studies from the United States and Brazil have demonstrated the use of next-generation sequencing (NGS) technologies to monitor genetic changes in circulating viral strains and identify novel mutations associated with increased transmission. This approach has been pivotal in detecting the introduction of new ZIKV strains in South and Central America and tracing their origins back to Southeast Asia. Despite these advances, the review also highlighted critical gaps in global surveillance, particularly in low-resource settings. Many endemic regions, especially in Africa and Southeast Asia, still face significant challenges in establishing robust surveillance networks. The lack of standardized reporting systems and real-time data sharing between countries has hindered timely responses to outbreaks, allowing for the spread of infections across borders [[70], [71], [72], [73], [74]].

The Dengvaxia vaccine, developed for dengue fever, has been approved in several countries, but concerns regarding its safety and efficacy, particularly in individuals who have not been previously infected with DENV, have limited its widespread use. Beyond Dengvaxia, several newer dengue vaccines have advanced significantly and are reshaping the landscape of dengue prevention. TAK-003 (Qdenga; Takeda) is a live-attenuated tetravalent dengue vaccine that has demonstrated efficacy across multiple dengue serotypes and has been approved for use in several countries for both seropositive and seronegative individuals. Unlike Dengvaxia, TAK-003 does not require prior dengue infection screening, enhancing its feasibility for large-scale immunization programs and routine use in endemic and high-risk settings. Its deployment offers a promising opportunity to reduce dengue incidence when integrated with existing vector control and surveillance strategies. Another important candidate is the Butantan dengue vaccine (Butantan-DV), a live-attenuated tetravalent vaccine developed in Brazil. Phase III clinical trials have shown encouraging efficacy and safety results, particularly in dengue-endemic populations. The Butantan-DV vaccine is being positioned for use in public-sector immunization programs in Latin America, with the potential to improve vaccine accessibility and regional self-sufficiency in dengue control. Together, these newer dengue vaccines address several limitations of earlier vaccine efforts and represent important tools for reducing disease burden. Progress has also been made in the development of vaccines against other arboviral diseases. Notably, chikungunya vaccines have reached advanced stages of development, with at least one vaccine achieving regulatory approval and initial implementation in selected populations, including older adults and individuals at increased risk of severe disease. The availability of a chikungunya vaccine marks a significant milestone, as control of this virus has historically relied almost exclusively on vector control and outbreak response measures [75,76].

However, the effectiveness of traditional methods, such as insecticide spraying, has been compromised by the development of insecticide resistance in key mosquito species. The review highlighted studies that documented resistance to pyrethroids and organophosphates in *Aedes aegypti* populations in countries such as India, Brazil, and Thailand, reducing the effectiveness of chemical control efforts. In response to growing resistance, alternative vector control strategies have been proposed and tested, including the release of genetically modified mosquitoes, Wolbachia-based biocontrol programs, and the use of bacterial larvicides. For instance, the release of genetically modified *Aedes aegypti* mosquitoes in Brazil, designed to carry a lethal gene that causes the death of mosquito larvae, has shown promising results in reducing mosquito populations in trial sites. Similarly, Wolbachia-infected mosquitoes have been successfully released in several countries, with studies indicating a significant reduction in dengue transmission by blocking the ability of mosquitoes to transmit the virus. Accination efforts have also been a critical part of the control strategy for some arboviral diseases. However, the availability and acceptance of vaccines remain limited. The Dengvaxia vaccine, developed for dengue fever, has been approved in several countries, but concerns regarding its safety and efficacy, particularly in individuals who have not been previously infected with DENV, have limited its widespread use. Similarly, the development of ZIKV vaccines has faced challenges, with several candidates still in the experimental phase and only a few reaching clinical trials [[77], [78], [79], [80], [81]].

The emergence of new and re-emerging arboviral diseases poses significant challenges for global health. Our review identified several potential threats, including the risk of the spread of viruses such as the West Nile virus (WNV) into new regions, and the emergence of novel arboviruses from animal reservoirs. In particular, the ongoing interaction between human populations and wildlife, exacerbated by deforestation and increased human encroachment into previously untouched ecosystems, are associated with an increased risk of spillover events, particularly at human–wildlife interfaces that may introduce novel pathogens to humans. Additionally, the rise of zoonotic transmission of arboviruses in wildlife populations, particularly in tropical forests, raises concerns about the capacity of current surveillance and control systems to detect and respond to these emerging viruses. For example, the potential for *Aedes* mosquitoes to host and transmit viruses such as Zika and chikungunya to new hosts, including other mammals, may create new pathways for human infection. The risk of these viruses undergoing genetic shifts in response to environmental pressures adds further complexity to the already challenging task of managing arboviral diseases. Our findings underscore the dynamic and evolving nature of arboviral diseases, characterized by their expanding geographic reach, evolving genetic diversity, and the continuous emergence of new threats. Despite significant advances in molecular diagnostics and vector control, challenges remain in managing these diseases effectively. Strengthening global surveillance networks, investing in innovative control strategies, and addressing the underlying environmental and socio-economic determinants of disease transmission are critical to mitigating the impact of arboviral infections on global public health. As arboviruses continue to evolve and adapt to changing conditions, a coordinated and proactive approach to surveillance, research, and intervention is essential to curbing the spread of these emerging infectious diseases [[82], [83], [84], [85], [86]], Arboviruses are maintained not only through urban human–mosquito transmission cycles but also through complex sylvatic transmission networks involving wildlife reservoirs and forest-associated vectors. Substantial evidence supports the existence of enzootic cycles for several major arboviruses, particularly yellow fever virus (YFV), dengue virus (DENV), Zika virus (ZIKV), and chikungunya virus (CHIKV), in which non-human primates (NHPs) play a critical role as amplifying or maintenance hosts. In these systems, arboviruses circulate independently of human populations, persisting in forest ecosystems and periodically spilling over into humans when ecological or behavioral conditions allow. Non-human primates have been extensively documented as key reservoirs for YFV in Africa and South America, where sylvatic transmission involves canopy-dwelling mosquitoes such as *Haemagogus* and *Sabethes* species. Human infections occur when individuals enter forested areas for occupational or recreational activities, facilitating spillover through so-called “bridging vectors” that feed on both wildlife and humans. Similar sylvatic maintenance has been described for ZIKV and DENV in parts of Africa and Southeast Asia, where serological and virological evidence indicates long-standing circulation among NHP populations independent of urban outbreaks [76,87].

**Fig. 1:**
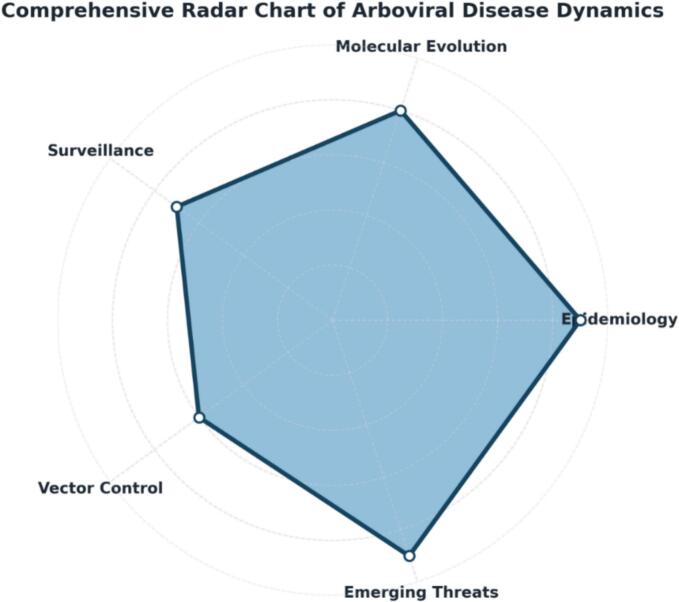
Multidimensional analysis of arboviral diseases: epidemiology, evolution, surveillance, control, and emerging threats.

**Fig. 2:**
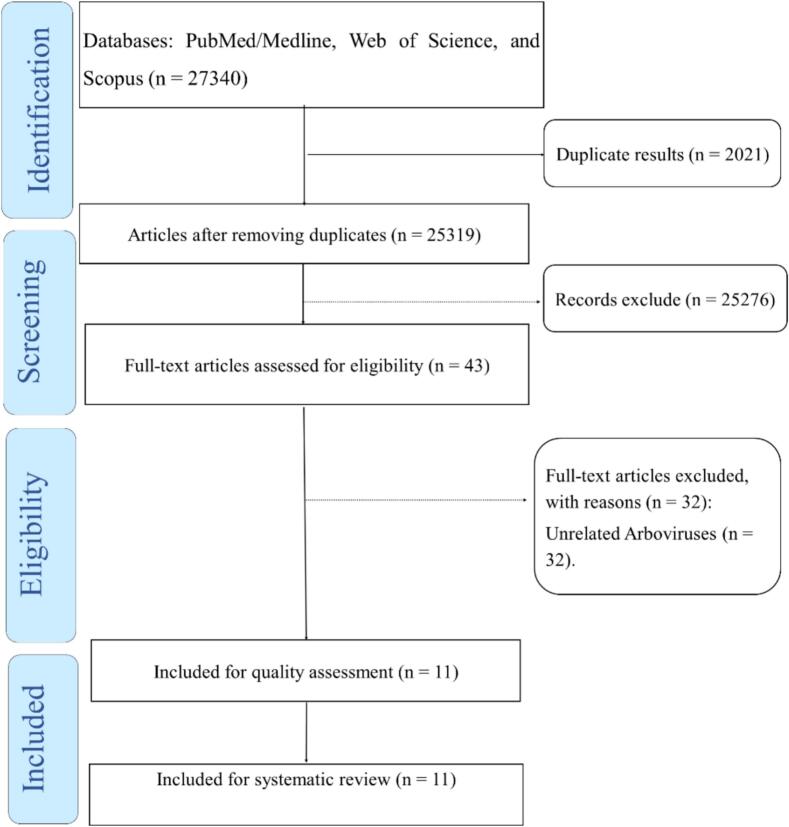
The review process based on the PRISMA flow diagram.

Bridging vectors, including *Aedes albopictus* and certain *Aedes* and *Culex* species, play a pivotal role in linking sylvatic and urban transmission cycles. These vectors exhibit opportunistic feeding behavior and ecological plasticity, allowing them to transmit viruses acquired from wildlife hosts to human populations. This ecological interface is particularly important in regions undergoing rapid deforestation, agricultural expansion, and urban encroachment, where increased contact between humans, wildlife, and vectors elevates spillover risk. Documented spillover events underscore the public health significance of sylvatic cycles. The re-emergence of yellow fever in Brazil has been closely linked to epizootics in NHP populations, which often precede human outbreaks and serve as early warning indicators of viral circulation. Similarly, experimental and ecological evidence suggests that ZIKV possesses the capacity to establish persistent sylvatic cycles in the Americas, raising concerns that eradication efforts focused solely on urban transmission may be insufficient. The potential for arboviruses to re-emerge from enzootic reservoirs complicates long-term control strategies and highlights the need for integrated surveillance systems that include wildlife monitoring. From a One Health perspective, recognizing the role of sylvatic reservoirs and animal–human transmission pathways are critical for understanding arbovirus persistence, geographic expansion, and re-emergence. Environmental change, biodiversity loss, and climate-driven shifts in vector distribution are likely to intensify interactions among wildlife, vectors, and human populations, thereby increasing the frequency of spillover events. Incorporating animal surveillance, ecological monitoring, and cross-sectoral collaboration into arboviral control programs is therefore essential for mitigating future outbreak risks and strengthening global preparedness [75,76,87,88].

(Table 1 and Fig. 1, Fig. 2).

**Table 1:** Summary of key findings on the epidemiology, evolution, and control of arboviral diseases.

Category	Key Findings	Implications/Challenges	Future Directions
Global Epidemiology	- Arboviral diseases are expanding in regions previously unaffected, including areas in Southeast Asia, Latin America, and Africa. - Rapid urbanization and climate change are the primary drivers of increased incidence.	- The spread of diseases like dengue, Zika, and chikungunya poses significant public health risks. - Increased vulnerability of low-income populations in urban areas.	- Enhanced global surveillance systems to track and predict outbreaks. - Targeted interventions in urban areas with rapid growth.
Vector Dynamics	- *Aedes aegypti* and *Aedes albopictus* are the primary vectors for several arboviruses. - Urbanization and inadequate waste management increase vector habitats.	- Increasing vector populations in urban centers, especially in tropical and subtropical regions. - Growing resistance to insecticides.	- Development of innovative vector control strategies, such as genetic modifications and Wolbachia-based interventions.
Molecular Evolution	- Arboviruses exhibit high genetic diversity, with mutations influencing transmission potential. - The evolution of new viral strains is linked to genetic adaptations in vectors.	- Genetic diversity complicates forecasting and control measures. - The emergence of new strains with altered virulence or transmission capacity.	- Continuous molecular surveillance to monitor viral evolution and detect new variants. - Focus on genetic markers that influence virulence and transmission dynamics.
Surveillance and Diagnostics	- Genomic surveillance tools (e.g., PCR, NGS) have advanced the identification and tracking of viral strains. - Geo-referencing and mobile technologies have improved real-time data collection.	- Limited diagnostic capacity in low-resource settings, especially in Africa and Southeast Asia. - Insufficient infrastructure and data-sharing for effective global monitoring.	- Strengthening diagnostic infrastructure in endemic regions. - Greater international collaboration for data sharing and reporting.
Control Strategies	- Current control strategies include insecticide use, mosquito genetic modification, and vaccine development. - Insecticide resistance has emerged as a significant challenge.	- Insecticide resistance limits the effectiveness of traditional vector control. - Vaccine coverage is limited, and current vaccines face safety concerns (e.g., Dengvaxia).	- Development of broad-spectrum vaccines. - Scaling up genetically modified mosquito programs and Wolbachia interventions.
Emerging Threats	- Zoonotic spillover events have increased, with new arboviruses emerging from animal reservoirs. - Environmental changes (e.g., deforestation, urban expansion) are contributing to new viral outbreaks.	- New viruses, such as Zika and CHIKV, present unpredictable public health challenges. - Increased risk of spillover events from wildlife to human populations.	- Further research into the ecological factors driving viral spillover. - Focus on preventing spillover through environmental protection and surveillance of animal reservoirs.
Climate Change Impact	- Climate change has been associated with changes in vector habitat suitability, which may contribute to the expansion of transmission areas. - Temperature, humidity, and rainfall patterns significantly impact arbovirus transmission.	- Changes in climate conditions may lead to longer transmission seasons and increased outbreaks. - Difficulty in predicting outbreak patterns due to fluctuating climatic conditions.	- Implementing climate-based prediction models for outbreak forecasting. - Development of adaptive vector control strategies responsive to changing climatic conditions.
Public Health Implications	- The increased spread of arboviral diseases highlights the need for comprehensive public health strategies. - Climate adaptation strategies are essential to reduce future risks.	- Urban health systems in developing countries are ill-prepared to manage the burden of arboviral diseases. - The disparity in health infrastructure exacerbates disease outcomes.	- Strengthening public health infrastructure, especially in urban and low-resource settings. - Integrating climate adaptation into public health policies.

## 4 Discussion

The increasing global incidence of arboviral diseases represents a significant public health challenge that necessitates a multifaceted approach to surveillance, control, and prevention. This systematic review has synthesized recent findings on the epidemiology, molecular evolution, vector dynamics, and control strategies of arboviruses, highlighting the key drivers of their emergence and spread. Our findings underscore the dynamic and evolving nature of these infections, which are influenced by a range of environmental, social, and biological factors. In this discussion, we contextualize these findings within broader global health trends and offer insights into the ongoing challenges and opportunities for controlling these diseases. The expansion of arboviral diseases beyond traditional endemic regions has been a defining feature of recent outbreaks, as evidenced by the increasing number of dengue, Zika, chikungunya, and yellow fever cases in areas previously unaffected by these pathogens. Our review indicates that climate change, urbanization, and global travel are consistently associated with the geographic expansion of arboviral diseases. Changes in temperature, rainfall patterns, and humidity have altered vector habitat suitability, enabling *Aedes aegypti* and *Aedes albopictus* mosquitoes to thrive in regions where they were previously not present. These vectors are highly adaptable and capable of surviving in diverse environmental conditions, making them particularly adept at establishing new populations in urban and peri-urban environments. The role of urbanization in facilitating the spread of arboviral diseases cannot be overstated. In rapidly growing cities, the proliferation of standing water due to inadequate waste management and poor sanitation provides ideal breeding sites for mosquitoes. As urban centers continue to expand, especially in the Global South, the risk of large-scale arboviral outbreaks increases. In this context, the disproportionate vulnerability of low-income populations, who often reside in poorly serviced areas, exacerbates the burden of these diseases. As our review highlighted, the increasing incidence of dengue and Zika in urban areas of Southeast Asia, Latin America, and Africa is a clear reflection of the interplay between rapid urbanization, environmental degradation, and insufficient public health infrastructure. Our findings also suggest that the seasonal nature of arboviral transmission, which is often driven by climatic factors, poses challenges for effective year-round surveillance and control. In regions with distinct rainy and dry seasons, outbreaks may exhibit cyclical patterns, with periods of higher transmission coinciding with peak vector populations. These seasonal fluctuations complicate the task of forecasting and managing outbreaks, underscoring the need for dynamic and adaptable surveillance systems that can respond to fluctuating transmission risks [62,63,89].

One of the most striking insights from our review is the significant genetic diversity observed among circulating strains of arboviruses. Molecular and phylogenetic analyses have revealed that these viruses are highly dynamic, with frequent genetic mutations contributing to their evolutionary success. This genetic diversity is particularly evident in the case of DENV, ZIKV, and CHIKV, where distinct viral serotypes and lineages are continually emerging. The ability of arboviruses to evolve rapidly in response to changing environmental pressures, such as increased vector competence or host immune responses, is a key factor in their persistence and spread. In particular, the genetic adaptations observed in ZIKV, which include mutations in the *E* protein that enhance vector competence, highlight the virus's ability to overcome ecological barriers and expand its range. These mutations are believed to contribute to the virus's increased infectivity in *Aedes albopictus*, a vector species that was previously less competent for ZIKV transmission. Similarly, the mutation rate observed in CHIKV, which has led to the emergence of novel strains with altered transmission dynamics, underscores the ongoing threat of viral evolution to public health control efforts. The evolutionary patterns of these viruses suggest that their adaptation is not only driven by genetic mutations but also by interactions with their vectors and hosts. Co-evolution between arboviruses and their mosquito vectors has led to the emergence of new viral strains with increased transmission potential. For example, the interplay between *Aedes* mosquitoes and DENV has resulted in the selection of viral strains that are more efficient at infecting and replicating within mosquito populations. This evolutionary process makes it difficult to predict future viral outbreaks, particularly as new genetic variants may exhibit altered transmission characteristics or even cross-species infectivity [[90], [91], [92]].

Advancements in surveillance and diagnostic technologies have significantly improved our ability to detect and respond to arboviral outbreaks. The use of molecular tools, such as PCR and next-generation sequencing (NGS), has revolutionized the identification of viral strains and the monitoring of viral evolution. Genomic surveillance, in particular, has enabled researchers to track the spread of arboviruses across borders and predict the emergence of new variants. The ability to conduct real-time sequencing of viral genomes has provided valuable insights into the mutation rates of these viruses and helped to identify potential markers of increased transmissibility or virulence. While these technological advances have greatly enhanced our understanding of arboviral transmission, challenges remain in implementing effective surveillance systems, particularly in resource-limited settings. As our review highlights, many endemic regions still lack the infrastructure and capacity to conduct widespread molecular surveillance. The absence of standardized reporting systems and limited data sharing between countries further hinders global efforts to monitor arboviral outbreaks. This is particularly problematic in Africa, where many countries lack adequate diagnostic facilities and have limited access to advanced molecular tools. In addition to improving diagnostic accuracy, the integration of geo-referencing tools and real-time data collection into surveillance systems has enhanced our ability to monitor and predict outbreaks. The use of mobile technologies, GIS mapping, and integrated health data systems can provide early warnings of potential outbreaks, allowing for targeted interventions before transmission reaches critical levels. However, the success of these systems depends on adequate training, funding, and international collaboration to ensure that data is collected consistently and shared transparently [[93], [94], [95]].

Despite advances in diagnostic technologies and surveillance methodologies, the effective implementation of arboviral surveillance and control strategies is strongly shaped by health-system capacity, governance structures, and broader socio-political contexts. In many endemic and at-risk regions, limited healthcare infrastructure, shortages of trained personnel, and constrained laboratory capacity impede timely case detection, reporting, and response. These challenges are particularly pronounced in low- and middle-income countries, where competing health priorities and fragile health systems reduce the ability to sustain routine surveillance outside of outbreak periods. Financing constraints represent a major barrier to scalable and sustainable arboviral control. Surveillance systems, vector control programs, and vaccination initiatives often rely on short-term or externally funded projects, which limits continuity and long-term planning. Inconsistent funding can result in fragmented data collection, delayed outbreak responses, and reduced effectiveness of control interventions. Moreover, the costs associated with advanced molecular surveillance, including genomic sequencing and data management, may be prohibitive in settings lacking dedicated public health investment. Data-sharing and coordination barriers further undermine the effectiveness of surveillance efforts. Fragmented reporting systems, lack of standardized indicators, and limited interoperability between human, animal, and environmental health databases hinder timely information exchange across sectors and administrative levels. Cross-border data sharing is particularly challenging, despite the transnational nature of arboviral transmission. Strengthening regional and global coordination mechanisms is therefore essential for early warning, risk assessment, and coordinated response to emerging outbreaks [96].

From a governance perspective, One Health frameworks offer a valuable approach for operationalizing integrated arboviral surveillance and control by linking human health, animal health, and environmental sectors. However, translating One Health principles into practice requires clear institutional mandates, intersectoral coordination mechanisms, and political commitment. Similarly, urban governance plays a critical role in arboviral control, as local authorities influence land use planning, water management, housing quality, and waste disposal key determinants of vector breeding and human exposure. Weak urban governance and informal settlement growth can undermine even well-designed technical interventions. Addressing arboviral threats therefore requires not only technological and biomedical solutions but also sustained investment in health systems, governance capacity, and cross-sectoral collaboration. Integrating surveillance and control strategies within broader health-system strengthening efforts and urban policy frameworks is essential to ensure that scientific advances translate into meaningful and equitable public health impact. The primary strategies for controlling arboviral diseases have historically centered on vector control and vaccination. Vaccination should be viewed as a complementary component of an integrated arboviral disease-control framework rather than a standalone intervention. While vaccines can substantially reduce disease incidence and severity, their effectiveness is maximized when combined with robust vector control, molecular and syndromic surveillance, and environmental management strategies. Surveillance systems remain essential for identifying high-risk populations, guiding vaccine deployment, and monitoring vaccine impact, particularly in the context of evolving viral lineages and shifting transmission patterns. In parallel, sustained vector control efforts are necessary to limit transmission of both vaccine-preventable and non-vaccine-preventable arboviruses, as well as to reduce the risk of spillover from sylvatic cycles. From a public health perspective, integrating vaccination into broader disease-control strategies enhances resilience against outbreaks, particularly in urban and peri-urban settings where arboviral transmission is intense. The combined use of vaccination, early-warning surveillance, and adaptive vector control provides a more sustainable and flexible approach to managing arboviral threats under conditions of climate change, urbanization, and increasing human mobility. This integrated strategy aligns closely with One Health principles and underscores the importance of coordinated, multisectoral responses to emerging and re-emerging arboviral diseases [97,98].

However, the growing problem of insecticide resistance in key mosquito populations, particularly in *Aedes aegypti*, has severely limited the effectiveness of chemical control methods. Our review highlights studies that document widespread resistance to pyrethroids and organophosphates in several regions, including South and Southeast Asia, Africa, and the Americas. This resistance threatens to undermine traditional vector control measures, particularly in urban areas where mosquito populations are dense and difficult to target. In response to insecticide resistance, novel control strategies have been proposed and tested, with mixed success. Genetic modification of mosquitoes, for example, has shown promise in reducing vector populations. The release of genetically modified mosquitoes designed to carry a lethal gene has successfully reduced mosquito populations in trial sites in Brazil and Malaysia, although challenges remain in scaling up these programs to achieve widespread impact. Similarly, Wolbachia-based mosquito releases, which involve infecting mosquitoes with a bacteria that renders them incapable of transmitting viruses, have been successfully implemented in several countries, including Australia, Vietnam, and Indonesia. These strategies have demonstrated significant reductions in dengue transmission in pilot sites, but their long-term sustainability and scalability remain uncertain. Vaccination remains a cornerstone of arboviral control, but its application is limited by the availability of vaccines and the challenges associated with their deployment. The Dengvaxia vaccine for dengue fever, although approved in several countries, has faced significant concerns regarding its safety in individuals who have not been previously infected with DENV. The risk of severe outcomes in seronegative individuals has led to the suspension of the vaccine in some countries, highlighting the need for further research to ensure the safety and efficacy of vaccines. In contrast, the development of a ZIKV vaccine remains in the early stages, with several candidates still undergoing preclinical testing. The emergence of new and re-emerging arboviruses, such as Zika and chikungunya, further complicates the landscape of vaccine development, as the need for polyvalent vaccines capable of protecting against multiple arboviruses becomes more pressing [80,99,100].

The ongoing emergence of new and re-emerging arboviruses poses significant challenges to global health. Our review highlights the increasing risk of spillover events, whereby arboviruses maintained in sylvatic transmission cycles involving wildlife reservoirs particularly non-human primates are transmitted to humans, often driven by deforestation, urban expansion, climate change, and the activity of bridging mosquito vectors. As human populations encroach on previously undisturbed ecosystems, the risk of encountering new zoonotic diseases increases. The spillover of viruses like Zika, West Nile virus (WNV), and various alphaviruses from wildlife to human populations has the potential to create new epidemiological and transmission dynamics that are difficult to predict. Furthermore, the increasing genetic diversity of arboviruses makes it difficult to anticipate how these viruses will evolve in response to ecological pressures, treatment interventions, or vaccine development. For instance, the rapid mutation rates of ZIKV and CHIKV complicate efforts to design effective vaccines and control measures, while the emergence of new vector species capable of transmitting these viruses further complicates their management [60,101,102].

### 4.1 Conclusion and future directions

This review has highlighted the complexities of understanding and controlling arboviral diseases, which are influenced by a combination of epidemiological, ecological, genetic, and social factors. While significant progress has been made in understanding the molecular evolution and transmission dynamics of these viruses, much work remains to be done. Strengthening global surveillance systems, improving diagnostic tools, and investing in innovative vector control strategies are critical to mitigating the public health threat posed by these diseases. Additionally, future research should focus on the development of broad-spectrum vaccines, addressing the challenges of insecticide resistance, and understanding the role of environmental changes in shaping the evolution of arboviruses. The fight against arboviral diseases requires a coordinated, multi-disciplinary approach that involves collaboration between public health authorities, researchers, and local communities. By focusing on early detection, rapid response, and sustainable control measures, we can better prepare for the next wave of emerging arboviral threats and ultimately reduce the global burden of these diseases [67,103,104].

## Consent for publication

Not applicable.

## Ethics approval and consent to participate

Not applicable.

## Funding

This research received no specific grant from any funding agency in the public, commercial, or not-for-profit sectors.

## Authors' contribution

E.A. has conducted all parts of the study, including design, execution, and writing the manuscript.

## Declaration of competing interest

The authors declare no competing interests.

## Data Availability

All data generated or analyzed during this study are included in this published article.

## Appendix A Supplementary data

Supplementary material Image 1
